# The eBioKit, a stand-alone educational platform for bioinformatics

**DOI:** 10.1371/journal.pcbi.1005616

**Published:** 2017-09-14

**Authors:** Rafael Hernández-de-Diego, Etienne P. de Villiers, Tomas Klingström, Hadrien Gourlé, Ana Conesa, Erik Bongcam-Rudloff

**Affiliations:** 1 SLU-Global Bioinformatics Centre, Department of Animal Breeding and Genetics, Swedish University of Agricultural Sciences, Uppsala, Sweden; 2 International Livestock Research Institute (ILRI), Nairobi, Kenya; 3 KEMRI-Wellcome Trust Research Programme, Kilifi, Kenya; 4 Centre for Tropical Medicine and Global Health, University of Oxford, Oxford, United Kingdom; 5 Genomics of Gene Expression Lab, Centro de Investigación Príncipe Felipe, Valencia, Spain; 6 Microbiology and Cell Science Department, Institute for Food and Agricultural Sciences, University of Florida, Gainesville, United States of America; Genome Quebec, CANADA

## Abstract

Bioinformatics skills have become essential for many research areas; however, the availability of qualified researchers is usually lower than the demand and training to increase the number of able bioinformaticians is an important task for the bioinformatics community. When conducting training or hands-on tutorials, the lack of control over the analysis tools and repositories often results in undesirable situations during training, as unavailable online tools or version conflicts may delay, complicate, or even prevent the successful completion of a training event. The eBioKit is a stand-alone educational platform that hosts numerous tools and databases for bioinformatics research and allows training to take place in a controlled environment. A key advantage of the eBioKit over other existing teaching solutions is that all the required software and databases are locally installed on the system, significantly reducing the dependence on the internet. Furthermore, the architecture of the eBioKit has demonstrated itself to be an excellent balance between portability and performance, not only making the eBioKit an exceptional educational tool but also providing small research groups with a platform to incorporate bioinformatics analysis in their research. As a result, the eBioKit has formed an integral part of training and research performed by a wide variety of universities and organizations such as the Pan African Bioinformatics Network (H3ABioNet) as part of the initiative Human Heredity and Health in Africa (H3Africa), the Southern Africa Network for Biosciences (SAnBio) initiative, the Biosciences eastern and central Africa (BecA) hub, and the International Glossina Genome Initiative.

This is a *PLOS Computational Biology* Education paper.

## Introduction

High throughput technologies and next generation sequencing require the development of new methods to manage the data generated by researchers. It is therefore imperative that training in bioinformatics is available to educate experts as well as other researchers in order to allow them to plan their research and properly assert the true cost and effort to complete a project [1].

In developing countries, bioinformatics has been a strategic investment for many countries due to its positive contributions to other fields of life science as well as the comparatively low costs of the discipline. Equipping and running a bioinformatics teaching laboratory cost less than equipping and running a biology laboratory [2] and many developing or formerly socialist countries have access to trained professionals in advanced mathematics and/or computational science [3], which form the basis of the field when combined with biology. From a political perspective, an enhanced capacity in bioinformatics allows researchers to conduct advanced analysis inside the country to ensure that the immaterial property rights are retained within the country and can support the development of a national life science industry [3, 4, 5].

Initial efforts in developing countries have generated numerous hubs of excellence located in the bigger or more affluent countries, but smaller countries are following suit [6]. Furthermore, international networks such as H3ABioNet [7] are developing a network of expert hubs for bioinformaticians collaborating with each other to strengthen international collaboration in developing countries [8]. Extensive training is, however, necessary to provide to the research professionals necessary to populate these networks and analyze the virtual mountains of data generated by modern research [9, 10].

**Fig 1:**
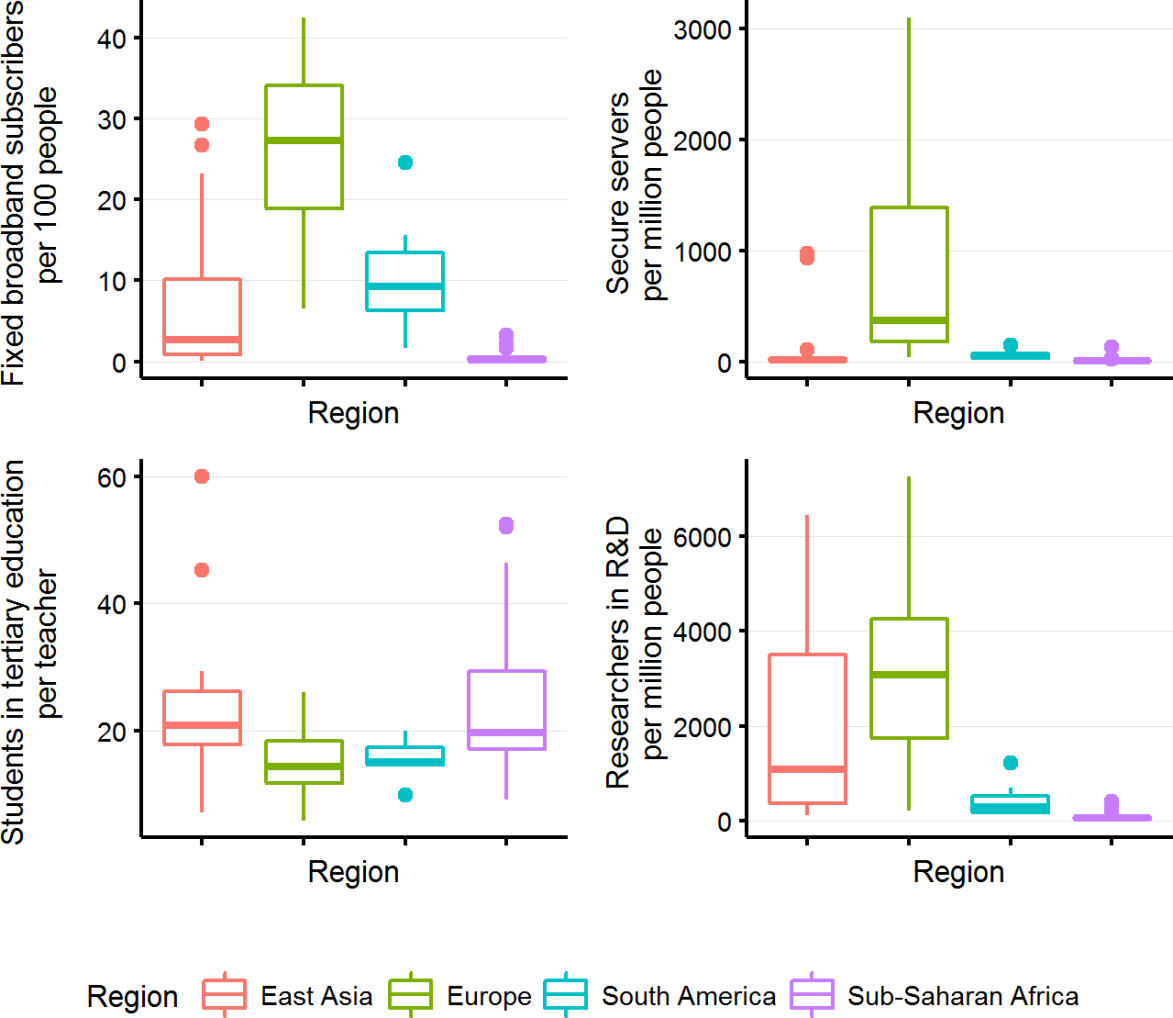
Box plots displaying access to technology and expertise in East Asia, Europe, South America, and sub-Saharan Africa. All data is calculated from the World Bank DataBank [15] (see S1 Table for for full data). The vertical lines extend from the 25th and 75th percentiles to the lowest/highest value that is within 1.5 times the distance between the 25th and 75th percentiles (the interquartile range). Data beyond the end of the lines are outliers and plotted as points. Data for fixed broadband internet subscribers (per 100 people) are per 2014; the number of secure servers by 2014 and other data is from a range of years from 2010 to 2015, depending on data availability (see S2 Table for summarized data per country).

The major challenges towards the creation or expansion of viable communities of bioinformaticians vary across the world based on the available resources and priorities within the education system. In the Western world, the chief concern regarding bioinformatics is a lack of trained professionals within the field who can conduct research and/or maintain infrastructures [11]. In the Asia-Pacific region, recruitment to the field is regarded as less of a problem, as young researchers perceive the field as an attractive career choice. Instead, chief concerns relate to the comparative lack of infrastructure in many countries [12]. These differences are also evident when comparing key development indicators for communications technology and research. Several of the most highly developed countries in East Asia are competitive with European nations in regards to the number of researchers per million people and student-to-teacher ratios in higher education. But only Japan and Singapore rank above the European median regarding the number of secure servers and fixed broadband subscriptions per capita (Fig 1). Furthermore, several of the nations in the region may place low on population-adjusted metrics but can still provide a high-quality infrastructure for universities and the growing middle class.

Such clusters of high capacity are, however, significantly rarer in South America and Africa [13] (Fig 1), which makes capacity building significantly more challenging, as logistics become a significant challenge when planning training sessions. Unreliable internet access, few local teachers, and a lack of suitable students are common issues and it is therefore important that training sessions are not delayed or disrupted, as the number of training opportunities involving international experts is limited. This makes it important that bioinformatics training in Africa is carefully planned and that measures are taken to ensure access to infrastructure suitable for bioinformatics [10]. As a result, African networks such as the H3ABioNet need to rely on using creative approaches to overcome these issues by seeking low latency alternatives and using portable devices that host data and tools and run independently of the network [14]. Key performance indicators provided by the World Bank DataBank [15] (Fig 1) and other resources indicate that internet connectivity [16, 17] as well as internet infrastructure are improving at a rapid rate in developing countries. Access to trained personnel in the form of researchers, technicians, and teachers is, however, increasing at a lower rate, indicating that even as internet connectivity technology improves, international support in the form of education and training will remain important.

The eBioKit was first developed in 2007 as a response to the lack of reliable and sufficient internet connections and the short time available to visiting researchers for conducting hands-on training at workshop or short courses. In many cases, utilization of large public databases of biomolecular data by the course software is required and valuable time is lost configuring locally provided computers to participants of the course. Furthermore, unforeseen delays are common even when a sufficient internet connection is available, as remote servers might be suddenly down or new software versions have been released that make on-site exercises fail or give different results than expected. This is an important burden, especially in these short courses provided by external lecturers, who have limited time to go over the teaching material.

Having experienced the difficulties for running the on-site courses in many world locations, it became clear that a solution was needed that would facilitate teaching without dependence on the internet and instabilities of the software. This has motivated the development of the eBioKit, a portable device for bioinformatics training (example installation: http://www.ebiokit.eu). In this paper, we present this teaching resource; describe the architecture, contents, and utilization of the system; and illustrate several projects in which the tool has been successfully used for teaching as well as research.

## Materials and methods

### Content of the platform

**Fig 2:**
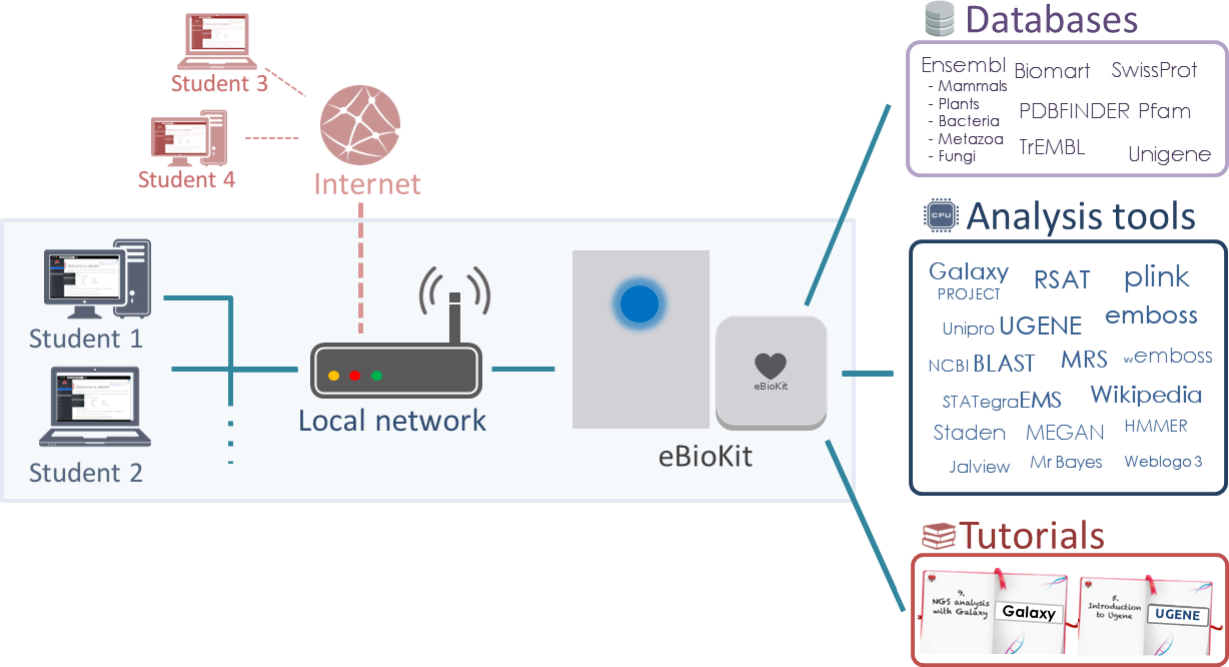
A typical installation of the eBioKit on a local network. Students and researchers can access the tools, databases, and tutorials installed in the eBioKit using the assigned local internet protocol (IP) address or a known uniform resource locator (URL). The eBioKit includes some administration tools that simplify the setting up of the platform on new networks. Additionally, if network configuration allows, the eBioKit services can also be accessed from external networks.

The eBioKit is a portable bioinformatics educational platform, the main purpose of which is to significantly reduce dependence on the internet by offering locally a wide range of services and repositories widely used in genomic research as well as documentation and material for training in their use (Fig 2). Local availability and portability are key elements in making the eBioKit an excellent educational tool in places with limited infrastructure.

There are 3 basic types of content in the eBioKit: databases, software, and tutorials. Research for both human and nonhuman model organisms is supported by the inclusion of Ensembl Mammals and Ensembl Genomes [18, 19], as both biomedical and environmental research is frequently relevant at targeted teaching locations. Protein functional analysis is supported by the inclusion of databases such as UniProtKB/Swiss-Prot [20], UniProtKB/TrEMBL [20], protein family database (Pfam) [21], and the Protein Data Bank [22]. In addition to databases, tools for sequencing homology search, protein structure prediction, next-generation sequencing (NGS) data analysis, functional annotation, and genome-wide association studies (GWASs) among others are included to give support to any bioinformatics discipline. Some of the services installed on the latest version of the eBioKit are listed in Table 1, and a more complete description can be found at http://www.ebiokit.eu/information. Moreover, many other popular bioinformatics tools and software utilities are also distributed on the eBioKit as downloadable resources or as part of comprehensive collections of analysis tools such as the Chipster platform [23] or generic model organism database (GMOD) in a Box [24], both available as virtual machine images.

**Table 1:** A selection of services installed on the eBioKit.

Service name	Type	Access				Version
		CL	DW	WB	GT	
Chipster [23]	Image		×			-
EMBOSS [25]	Tool	×			×	6.6.0
Ensembl^1^ [18, 19]	Database			×		Releases 75 and 22
BioMart [26]	Database			×		0.7
Galaxy [27]	Tool			×		July 2014
GMOD in a Box [24]	Image		×			2.05
Jalview [28]	Tool	×	×	×		2.8.1
MRS [29]	Database	×		×		6.0.3
NCBI Blast^2^ [30]	Tool	×		×	×	2.2.26 and 2.2.29+
PLINK [31]	Tool		×	×		v1.07
RSAT [32]	Tool	×	×	×		October 2014
STATegra EMS [33]	Tool			×		0.6r1
WebApollo [34]	Tool			×		v1.0.3
wEMBOSS [35]	Tool			×		v2.2.1

^1^Ensembl Mammals (release 75), Bacteria (release 22 [r22]), Fungi (r22), Metazoa (r22), Plants (r22) and Protists (r22).

^2^NCBI Blast and NCBI Blast+.

**Abbreviations:** CL, command-line; DW, downloadable resource; EMBOSS, the European Molecular Biology Open Software Suite; EMS, Experiment Management System; GMOD, Generic Model Organism Database; GT, Galaxy tool; MRS, Maarten’s Retrieval System; NCBI, National Center for Biotechnology Information; RSAT, Regulatory Sequence Analysis Tools; WB, web-based; wEMBOSS, web-interface to EMBOSS

### User interface

**Fig 3:**
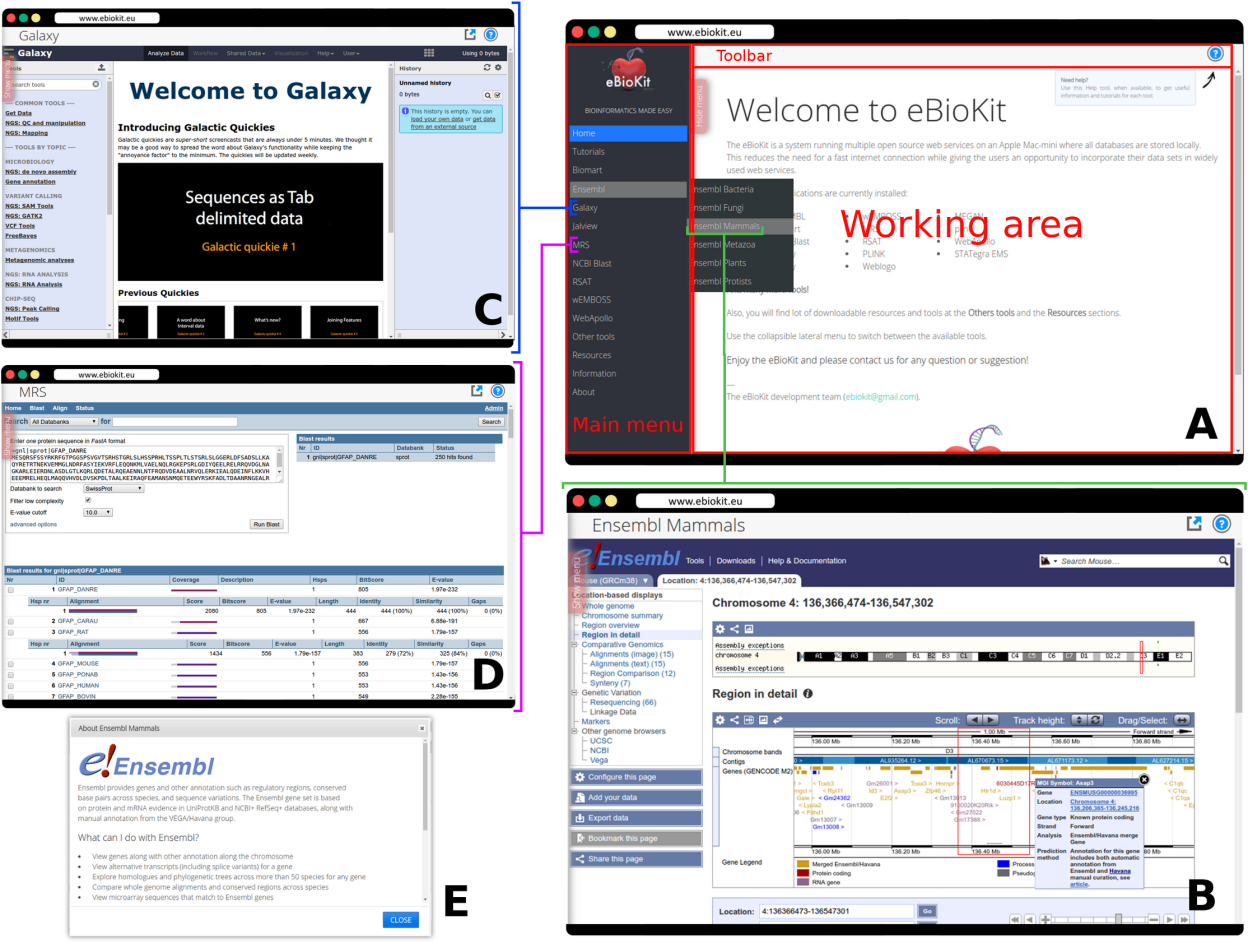
Web interface for the eBioKit. (A) The arrangement of the components that compose the web interface. Users can easily switch between the installed services on the eBioKit using the lateral menu. When users choose an option on the menu, the working area is replaced by the corresponding service and the menu is hidden, allowing users to fully interact with the service. (B), (C), and (D) show the familiar web interface users see when working with Ensembl Mammals, Galaxy, and MRS, respectively, in eBioKit. With the upper toolbar, users can open the services on a secondary window and, more importantly, can get a description as well as download documentation and tutorials for the selected service (E).

The eBioKit is usually installed as a centralized service on the local network. Students can connect to the system by accessing to the local internet protocol (IP) address or a known uniform resource locator (URL) assigned to the eBioKit (Fig 2). The eBioKit has 2 basic access modes. The most common way is using a web browser of choice present in the student's computer. As depicted in Fig 3, the eBioKit website is divided into 2 main parts, the working area and the applications menu. Using this lateral menu, the students can switch between the installed tools and databases, and the content of the working area will be adapted to the selected service. Alternatively, students can connect via command-line interface using Secure Shell (ssh) on a terminal. This also gives the opportunity to train on command-line analysis tools that are not available with a graphical user interface and allows for flexibility in the definition of course contents and competences by the instructor, who can choose to include programming modules in the course material or simply teach web-/tool-based bioinformatics.

### Teaching material

**Fig 4:**
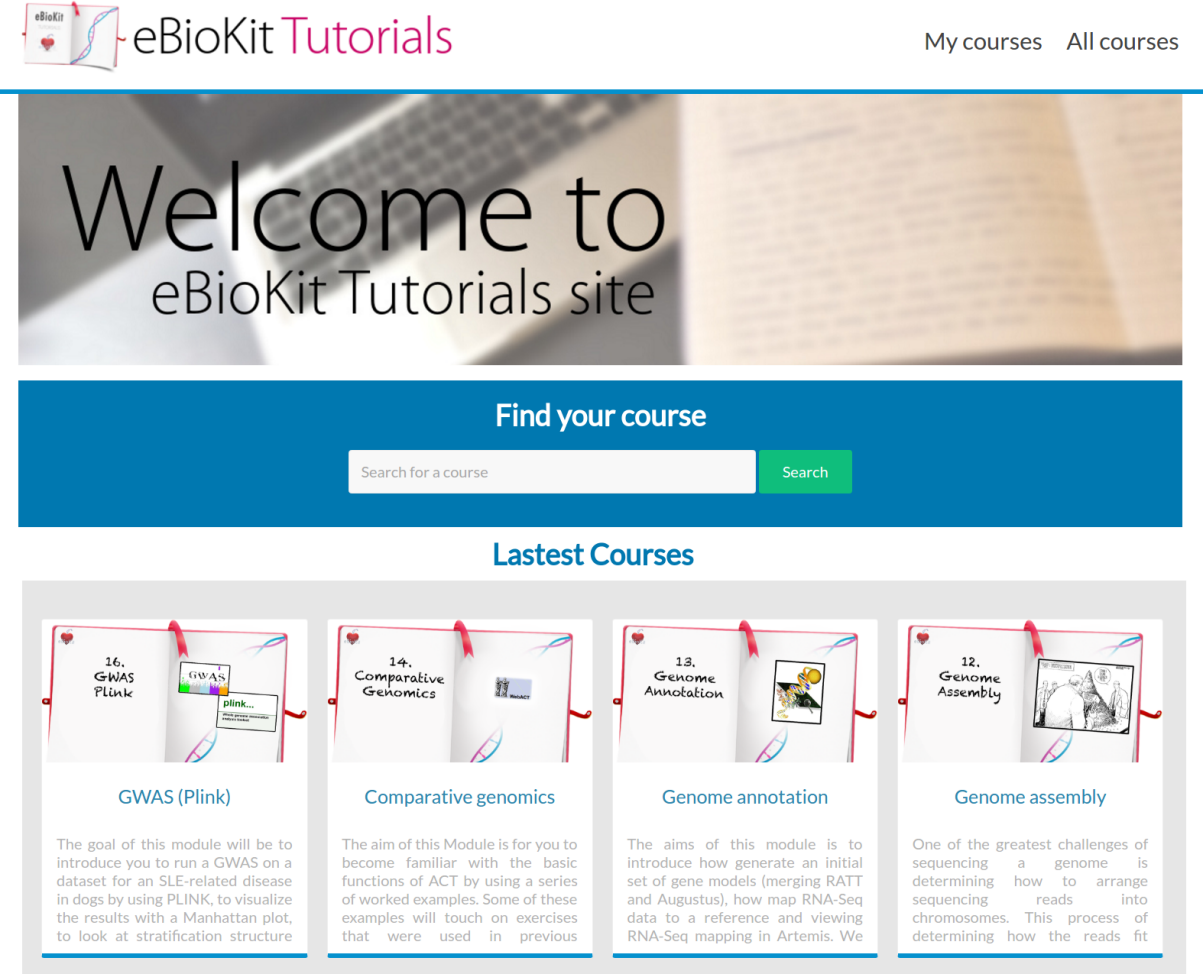
Entry page for the training portal in the eBioKit.

**Fig 5:**
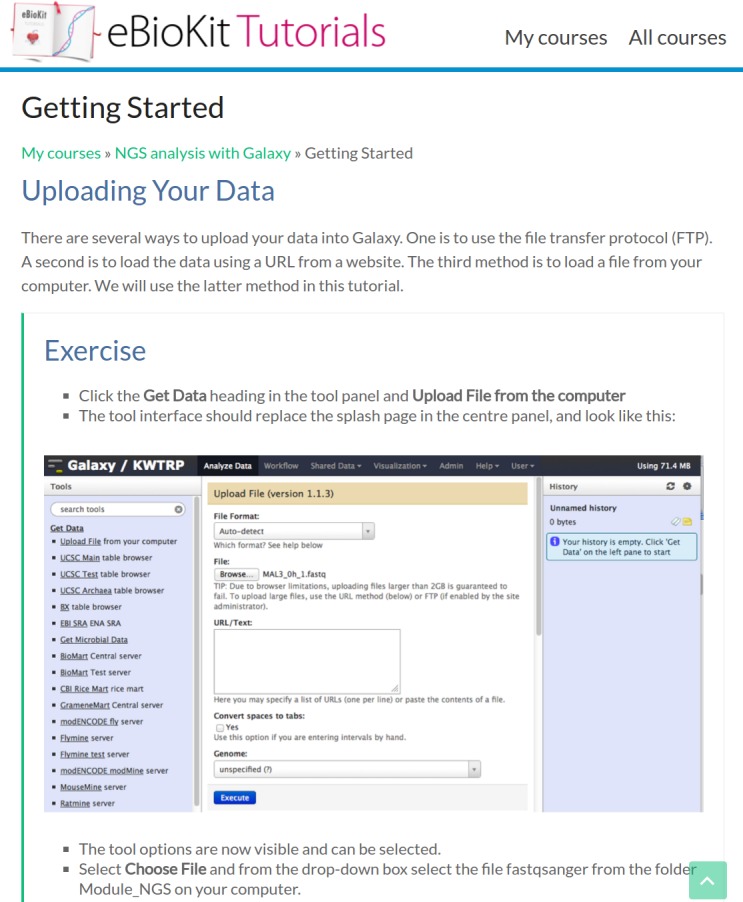
An example of a lesson in the eBioKit training portal. The image displays an extract of the “Getting started” lesson for the tutorial “next-generation sequencing (NGS) analysis with Galaxy.” During a tutorial, the students will find multiple exercises that allow them to put into practice the content learned.

Tutorials are a fundamental part of the eBioKit and are hosted on an e-learning platform in a unified environment, ensuring a cohesive learning experience (Fig 4). The included tutorials range from basic bioinformatics concepts to advanced topics such as high throughput sequencing analysis or GWAS. Tutorials are organized in courses, which are divided into lessons that usually correspond to a specific task for the student to accomplish such as building a reference genome or manipulating a dataset (Fig 5). All the required software and databases for each lesson are locally available, and data is often adapted to allow students to perform their analysis in a timely manner.

Most of the tutorials included in the eBioKit have been written and refined by our instructors over the years, but a special effort is being made to acquire new content from the community and adapt it for inclusion in the eBioKit. Tutorials are written in Markdown, a lightweight markup language that allows creating styled documentation, and most of them are available on the web-based Git repository GitHub for anyone to modify or reuse [36].

### System administration

**Fig 6:**
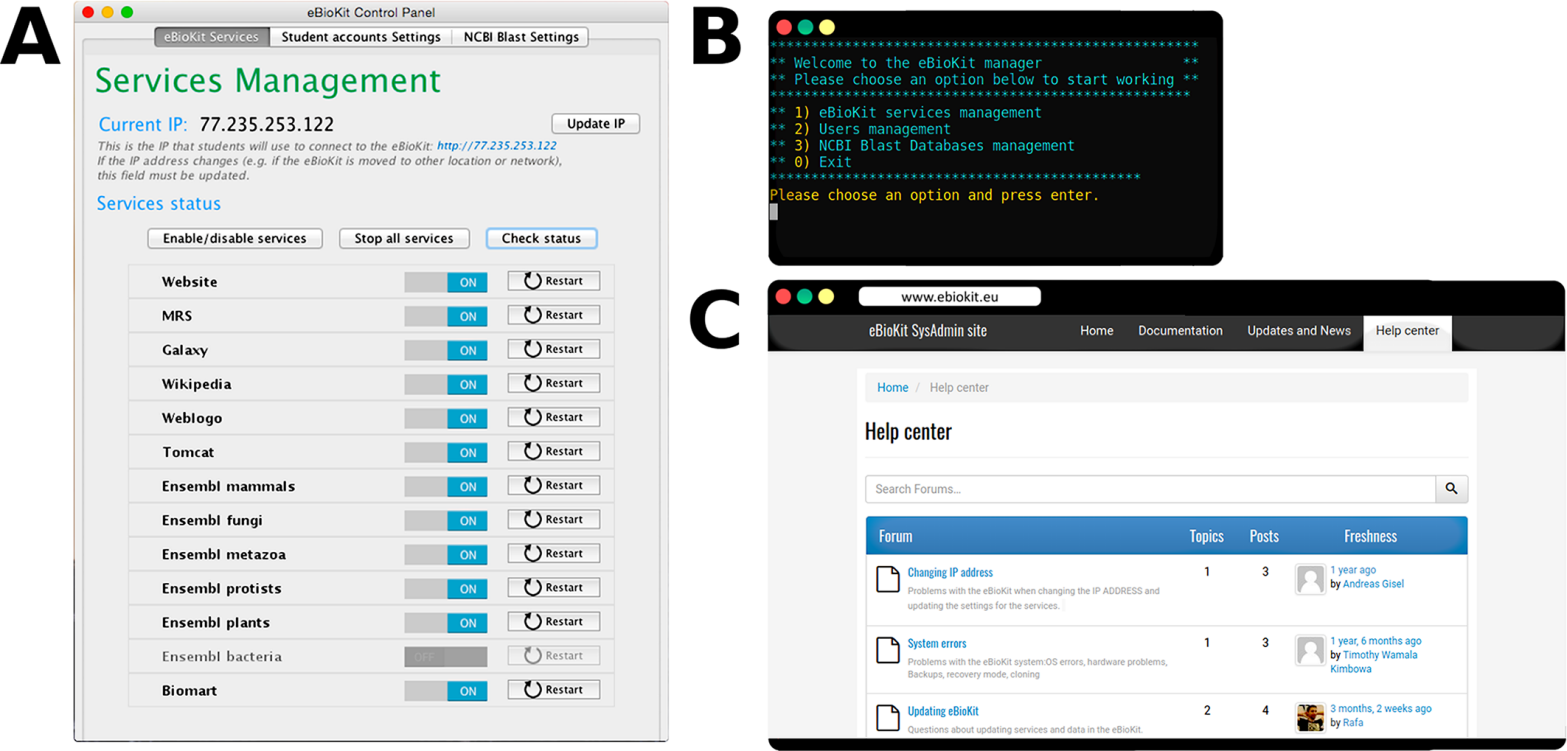
Tools for eBioKit administrators. (A) The eBioKit Control Panel desktop application. At the image, the section for services management displays the state for the installed services and includes some options for their configuration. Other sections provide access to user management and for NCBI Blast databases manipulation. This control panel is also available as a command-line utility, ideal for remote administration (B). Finally, the online help desk (C) contains news and updates for the eBioKit and introduces a communication channel between administrators and the eBioKit developers.

An important aspect for the reliable operation of the eBioKit is the administration of the system. As usual in this field, the administrators for the eBioKit must ensure the proper functioning of the installed services as well as provide users with support and keep the system updated and secure. For an easier administration, the eBioKit includes several tools that simplify some usual tasks in the management of services and users. These administration tools, which can be individually executed as command-line programs, are compiled in a Java application named "eBioKit Control Panel" that provides a user-friendly interface both as a desktop-based application and as a command-line application (Fig 6A and 6B). Moreover, an online help desk portal is maintained, where the administrators can get support directly from the developers as well as share their experiences or suggestions and find documentation, news, and other useful information related with the administration of the eBioKit (Fig 6C).

### Selection of hardware

Both computational and space requirements of many key bioinformatics tools are heavy and this turns portability into a complex objective to achieve. To address this issue, the eBioKit system has been historically built on Apple Mac Mini machines, which accomplish a brilliant balance of portability (the dimensions of the latest model are 197 x 197 x 36 mm and 1.2 kg of weight), computational capacity (up to 3.0 GHz dual-core Intel Core i7 and 16 gigabytes [GB] of random access memory [RAM]in the latest models) [37], and quality and reliable hardware.

In addition to the Mac Mini version, an alternative architecture using Mac Pro machines is available, slightly reducing the portability of the system (251 mm height, 167 mm diameter, and 5 kg of weight) but dramatically increasing the computational power (up to 3.5 GHz six-core Intel Xeon E5 and 64 GB in the latest models) [38].

**Fig 7:**
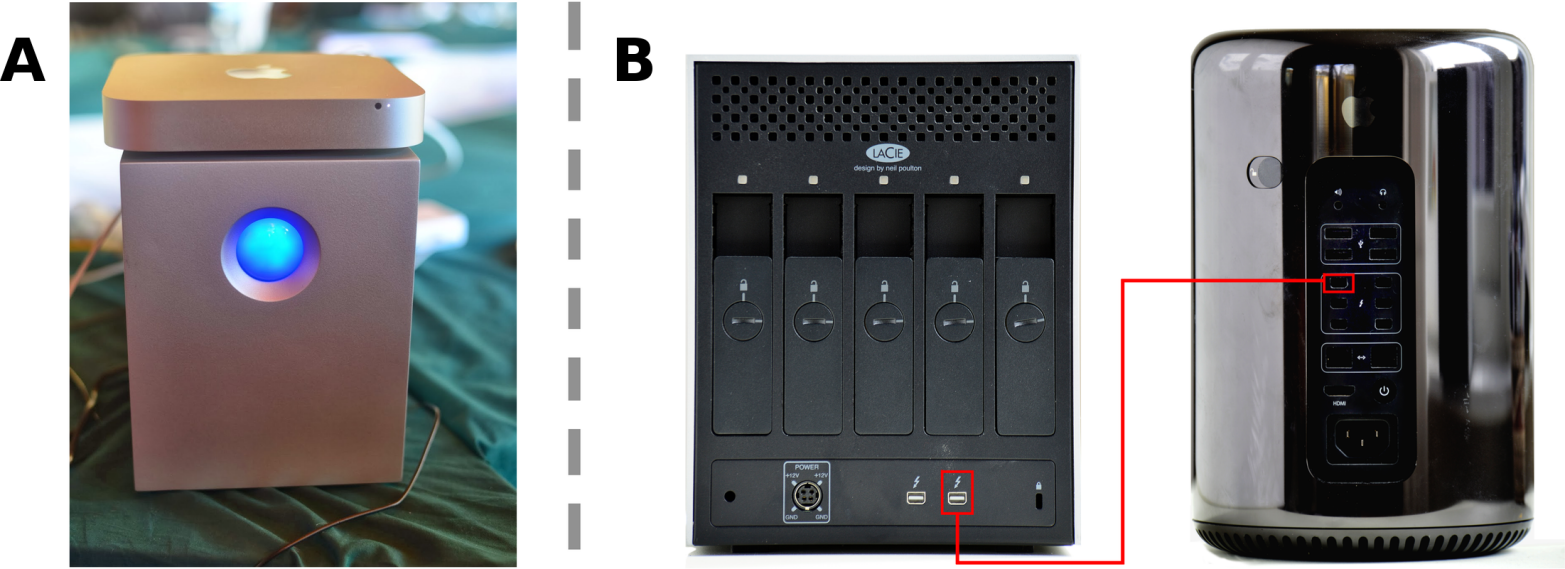
The 2 variants for the architecture of the eBioKit. (A) An eBioKit built on a Mac Mini with a 10-terabyte (TB) LaCie 5big Thunderbolt hard disk. (B) Detail for the Thunderbolt connection for the eBioKit Mac Pro version.

Storage supposes an added difficulty for portability and performance. The sizes of the biological resources installed on the eBioKit, such as the Ensembl Mammals databases, are in the range of several terabytes and increase with each new release. Nowadays, it is becoming easier to find on the market external storages in the multiple-terabytes range, most of them based on universal serial bus (USB) v3.0. For the eBioKit, the chosen storage solution was the LaCie 5big Thunderbolt (10 terabytes [TB] RAID0, 7,200 rpm, 173 x 220 x 196 mm and 9.9 kg), which takes advantage of the Thunderbolt port available on Mac machines (both in Mac Mini and Mac Pro versions), achieving a transfer rate of up to 700 megabytes [MBs] for read and write operations, independently [39] (Fig 7).

## Results

The eBioKit is distributed as an affordable and self-contained computing platform and database system containing up to 6 terabytes of biological data and software tools of relevance to bioinformatics researching, including the Ensembl database systems [18, 19], the European Molecular Biology Open Software Suite (EMBOSS) [25], Galaxy [27], National Center for Biotechnology Information (NCBI) Blast [30], and PLINK [31], which are made locally available through a unified web-based user interface.

From a teaching perspective, almost each tool or database installed in the platform includes a tutorial that introduces to its use. A total of 13 courses are currently available in the eBioKit. Courses encompass a wide range of bioinformatics disciplines ranging from basic bioinformatics tasks, the UNIX environment, and programming, to more advanced topics such as GWAS, RNA sequencing (RNA-Seq) analysis, genome assembly and annotation, and comparative genomics. S3 Table summarizes the content and the structure for the included courses.

**Fig 8:**
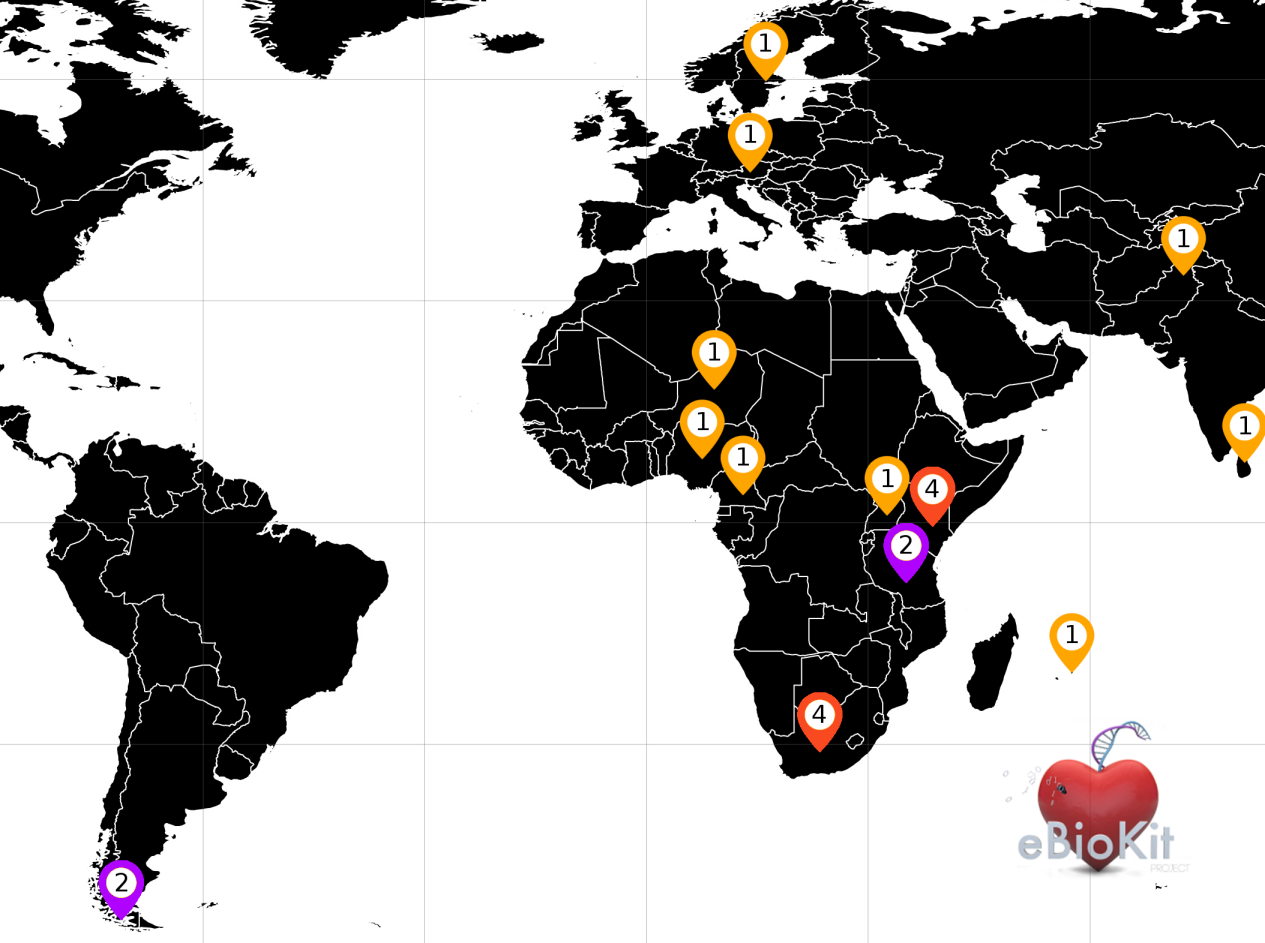
Global distribution for the eBioKit project. Numbers indicate the number of eBioKits deployed in each country. European institutions: Swedish University of Agricultural Sciences (SLU) (Sweden) and Medizinische Universität (Austria). South America: Instituto Antártico Chileno (INACH) and Universidad de Magallanes (Chile). Asian institutions: University of Colombo (Sri Lanka) and COMSATS (Pakistan). Africa: Centre de Recherche Medicale et Sanitaire (Niger), The International Institute of Tropical Agriculture (IITA) (Nigeria), University of Buea (Cameroon), Uganda Virus Research Institute (Uganda), BECA/ILRI (Kenya), Pwani University (Kenya), International Centre of Insect Physiology and Ecology (Kenya), Technical University of Kenya (Kenya), University of Dar es Salaam (Tanzania), Mikocheni Agricultural Research Institute (Tanzania), University of Mauritius (Mauritius), University of the Witwatersrand (South Africa), University of Pretoria (South Africa), University of Cape Town (South Africa), and SANBI South African National Bioinformatics Institute (South Africa).

A total of 24 training activities have been successfully organized during the last years with the help of the eBioKit in different research centres and universities across Europe, Africa, Asia, and South America in collaboration with international organisms such as the Pan African Bioinformatics Network (H3ABioNet) as part of the H3Africa initiative [15], the Southern Africa Network for Biosciences (SAnBio) [40], the Biosciences eastern and central Africa-International Livestock Research Institute (BecA-ILRI) hub [41], and the International Glossina Genome Initiative [42]. Moreover, the system has been acquired by many of those institutions as part of their computing facilities (Fig 8), allowing researchers to conduct bioinformatics-based research without having access to a reliable internet connection.

Concerning system performance, the version of the eBioKit built on Apple Mac Mini machines with 16 GB of RAM has been successfully used for courses with up to 25 students working in parallel on the courses included in the platform. This version may not be recommended for large NGS analysis work. On the other hand, the Mac Pro–based version with 32 GB RAM allows up to 40 students to attend to a course and work simultaneously. In spite of the system having not been benchmarked for intensive work yet, it is known that the system is routinely used as an analytical resource by some of the project partners.

## Discussion

The key benefit of the eBioKit is that it provides a controlled and reliable environment for bioinformatics. Tools and databases are constantly updated as research in the field progresses and results as well as user interfaces may change as new versions are released. For a researcher conducting training, this may present major issues as students are confused or critical components fail, which prevent the students from properly completing their tasks. Unfortunately, the costs of failure in training are the highest in the areas that can least afford to pay them.

In a developed region with access to local teachers and high-quality infrastructure, replacements and extra trainers can in most cases be brought in to complete the training goals. In a resource-constrained setting, teachers are often brought in from afar, which puts strict time limits on training as tickets are booked in advance, repairs of infrastructure often take longer, and access to local experts who can quickly solve issues or help complete the training is not always available.

The eBioKit is based on standardized and compact hardware, which makes it easy for trainers to prepare in advance. As all software is either open source or at least free for academic use, a trainer can, when necessary, purchase the necessary hardware and clone the eBioKit content, copying all necessary tools and data to the new server. The server is then brought along by the trainer to the training location and the students access the eBioKit through the local area network and work directly on the server, which avoids installation issues, unforeseen updates of web services, or failures in the local infrastructures. Upon completion of the training, the eBioKit can then either be brought back for future training sessions or left behind for use by local researchers or trainers.

## Conclusions

Bioinformatics has gradually established itself as an essential discipline for many life sciences and the consequent demand of qualified researchers has boosted the emergence of new educational approaches. Providing training in bioinformatics is challenging from many perspectives. The growth in the volume of biological data, the multidisciplinary skills required for students, and the necessary computing infrastructure as well as the constant development of methods and tools are some of the hurdles that must be tackled when setting up and maintaining an effective teaching infrastructure.

Several solutions have been developed in the last years for educational purposes in bioinformatics, but many of them demonstrate a lack of sustainability and a strong dependence on the internet, computing capacity, and third-party services, which usually lead to outdated tools and frustrating errors. We have developed a stand-alone and portable educational platform that allows the deployment of new educational resources together with the bioinformatics tools and databases needed for sustainable reuse of the teaching materials.

The eBioKit has been conceived to be a robust, user-friendly, and easy-to-manage teaching tool for courses and workshops and has demonstrated itself to be a valuable resource for institutions, universities, research centers, or schools that would like to start teaching bioinformatics or even provide bioinformatics capabilities for their groups. The platform is based on open source and open access licenses that ensure its availability and distribution and can be ordered directly to the developer team at no cost except those derived from the purchase of the necessary hardware to run the system (i.e., the Mac Mini or Mac Pro machines) and transportation. The advantage of the eBioKit as a training platform is the fact that it has self-contained courses and tutorials, teaching both basic and advanced bioinformatics using software and databases installed locally on the platform.

The eBioKit is a live project in constant development, providing a responsive support for users and administrators as well as inspiring other projects [43, 44]. Each iteration of the project is, however, functioning as a stable stand-alone platform, allowing researchers to teach and use the platform without compatibility issues. This allows researchers to conduct projects and training sessions without spending valuable time or resources on recreating a functioning environment each time a new course or project is initiated. More information about how to order an eBioKit and how to contribute to the project as well as other frequently asked questions and tools for contacting the eBioKit team can be found at http://www.ebiokit.eu.

## Supporting information

S1 Table Worldwide distribution for internet access, access to secure internet servers, pupil-teacher ratio, researchers in research and development (R&D), and technicians in R&D by country. Source: The World Bank databank and others [13, 16, 17].
(XLSX)

S2 Table Summarized worldwide distribution for internet access, access to secure internet servers, pupil-teacher ratio, researchers in research and development (R&D), and technicians in R&D by country. Source: The World Bank databank and others [13, 16, 17].
(XLSX)

S3 Table Overview of the included courses in the eBioKit. Each course in the eBioKit comprises several lessons, which cover popular topics in bioinformatics analysis and introduce the students to the usage of the software and databases locally installed.
(DOCX)
