# Supplementary material for: The eBioKit, a stand-alone educational platform for bioinformatics
Source: PLoS Comput Biol. 2017 Sep 14;13(9):e1005616. doi: 10.1371/journal.pcbi.1005616 (PMC5598936; doi:10.1371/journal.pcbi.1005616)
Supplement: S3 Table — Each course in the eBioKit comprises several lessons, which cover popular topics in bioinformatics analysis and introduce the students to the usage of the software and databases locally installed. (DOCX) [file pcbi.1005616.s003.docx]

| **Course name** | **Lessons** | **Topics** |
| --- | --- | --- |
| Introduction to UNIX | 2 | UNIX, PROGRAMMING, TERMINAL |
| Introduction to programming | 4 | UNIX, PROGRAMMING, TERMINAL, PERL |
| General sequence analysis using EMBOSS/wEMBOSS | 7 | SEQUENCE ALIGNMENT, PROTEIN ANALYSIS, VISUALIZATION, SEQUENCE ANALYSIS |
| Introduction to UGENE | 5 | VISUALIZATION, SEQUENCE ANALYSIS, SEQUENCE ALIGNMENT, PHYLOGENY, SEQUENCE ANNOTATION |
| NGS analysis with Galaxy | 3 | NGS ANALYSIS, QUALITY CONTROL, VISUALIZATION, SEQUENCE ALIGNMENT |
| Genome analysis with Artemis | 5 | PATHOGEN GENOMICS, VISUALIZATION, GENOME BROWSER, GENOME ANNOTATION |
| Mapping short reads | 3 | PATHOGEN GENOMICS, TERMINAL, NGS ANALYSIS, GENOME BROWSER, SEQUENCE ALIGNMENT |
| Genome assembly | 12 | DE NOVO ASSEMBLY, GENOME BROWSER |
| Genome annotation | 4 | SEQUENCE ANNOTATION, NGS ANALYSIS, RNA-SEQ, QUALITY CONTROL, VISUALIZATION |
| Comparative genomics | 4 | COMPARATIVE GENOMICS, NGS ANALYSIS, GENOME BROWSER |
| Genome-wide association study (Plink) | 2 | GENOME ANALYSIS, TERMINAL |
